# Changes of Structural and Functional Attention Control Networks in Subclinical Hypothyroidism

**DOI:** 10.3389/fnbeh.2021.725908

**Published:** 2021-10-29

**Authors:** Jingjing Yin, Lei Xie, DongXue Luo, Jinzhuang Huang, Ruiwei Guo, Yanmin Zheng, Wencan Xu, Shouxing Duan, Zhirong Lin, Shuhua Ma

**Affiliations:** ^1^Department of Radiology, The First Affiliated Hospital of Shantou University Medical College, Shantou, China; ^2^Department of Medical Imaging and Nuclear Medicine, Shantou University Medical College, Shantou, China; ^3^Laboratory of Medical Molecular Imaging, The First Affiliated Hospital of Shantou University Medical College, Shantou, China; ^4^Department of Nuclear Medicine, The First Affiliated Hospital of Shantou University Medical College, Shantou, China; ^5^Department of Endocrinology, The First Affiliated Hospital of Shantou University Medical College, Shantou, China; ^6^Department of Pediatric Surgery, The First Affiliated Hospital of Shantou University Medical College, Shantou, China

**Keywords:** subclinical hypothyroidism, attention, voxel-based morphometry, functional magnetic resonance imaging, brain function

## Abstract

**Objective:** This study aimed to explore the structural changes in patients with subclinical hypothyroidism (SCH) using voxel-based morphometry (VBM) and to investigate the altered attentional control networks using functional MRI (fMRI) during the performance of a modified Stroop task with Chinese characters.

**Methods:** High-resolution three-dimensional (3D) T1-weighted images and an fMRI scan were taken from 18 patients with SCH and 18 matched control subjects. The Montreal Cognitive Assessment Chinese-revised (MoCA-CR) and the Stroop task were used to evaluate the cognitive and attention control of the participants.

**Results:** Compared to controls, the VBM results showed decreased gray matter volumes (GMVs) in bilateral prefrontal cortices (PFCs, including middle, medial, and inferior frontal gyri), cingulate gyrus, precuneus, left middle temporal gyrus, and insula in patients with SCH. The fMRI results showed a distributed network of brain regions in both groups, consisting of PFCs (including superior and middle and inferior frontal cortices), anterior cingulate cortex (ACC), posterior cingulate cortex, and precuneus, as well as the insula and caudate nucleus. Compared to controls, the SCH group had lower activation of the above brain areas, especially during the color-naming task. In addition, the normalized GMV (nGMV) was negatively correlated with thyroid-stimulating hormone (TSH) level (*r* = −0.722, *p* < 0.001).

**Conclusion:** Results indicate that patients with SCH exhibit reduced GMVs, altered BOLD signals, and activation in regions associated with attention control, which further suggest that patients with SCH may have attentional control deficiency, and the weakened PFC–ACC–precuneus brain network might be one of the neural mechanisms. Negative correlations between nGMV and TSH suggest that TSH elevation may induce abnormalities in the cortex.

## Introduction

Thyroid dysfunction is a common endocrine disease that is manifested in four types, namely, hyperthyroidism, hypothyroidism, subclinical hyperthyroidism, and subclinical hypothyroidism (SCH). The SCH is characterized by abnormally elevated serum thyroid-stimulating hormone (TSH) levels, while the levels of serum free triiodothyronine (FT3) and free thyroxine (FT4) remain within the reference range (Cooper and Biondi, 2012). Many studies have shown, mainly using neuropsychological tests, that hypothyroidism or SCH is related to cognitive impairments, such as attention, memory, psychomotor, and executive functions (Resta et al., 2012; Pasqualetti et al., 2015; Yuan et al., 2020). Using PET, Bauer et al. (2009) and Constant et al. (2001) have found that patients with hypothyroidism exhibit lower regional activity and reduced regional cerebral blood flow in brain areas related to cognitive function. A series of task-based functional MRI (fMRI) studies also reveal task-induced deactivation in the default mode network or reversible alterations of the brain during working memory tasks (Zhu et al., 2006; He et al., 2011). Kumar et al. showed that the alterations of intrinsic resting-state functional connectivity in the somatomotor and right frontal-parietal attention networks may be one of the reasons for mild impairment of motor, working memory, attention, and executive cognitive functions in patients with SCH (Kumar et al., 2018).

Although researchers have tended to discuss attention and other cognitive functions (e.g., working memory) separately, the development of cognitive neuroscience has clearly shown that they are interdependent and share many of the same neural bases (Taylor et al., 1997; MacDonald et al., 2000). Several studies have found that decreases in working memory efficiency may be attributed to the possible decline of attentional control (Taylor et al., 1997; Wagner, 1999; MacDonald et al., 2000). Flexible cognitive control over our behavior is a key part of human intelligence. Attentional control is a mechanism by which the brain attempts to limit its processing of task-related information. In short, it is responsible for inhibitory functions. Our previous study showed that the spatial working memory of patients with SCH was impaired along with abnormally low activity in the right dorsolateral prefrontal cortex (PFC) and right posterior parietal lobe (Yin et al., 2013). All of these warrant further study of the attentional function of SCH. To identify the changes of patients with SCH in the neural substrates of the attentional control, we used a Stroop task because it is a powerful and simple task (Taylor et al., 1997).

The voxel-based morphometry (VBM) by Ashburner and Friston can quantitatively analyze and evaluate the structural changes of the brain by measuring the volume changes of gray matter (GM) (Ashburner and Friston, 2000). Differences in brain histomorphology are evaluated by calculating the density or volume of GM in the whole brain. Some investigators have studied the changes of the brain structure in patients with thyroid dysfunction (Singh et al., 2013; Quinque et al., 2014; Zhang et al., 2014). Singh et al. indicated significant morphological differences in the primary motor cortex, cerebellum, frontal, temporal, and occipital gyrus in patients with hypothyroidism (Singh et al., 2013). Thus, it is necessary to conduct a further study.

In this study, we used neuropsychological testing combined with task-based fMRI and VBM to identify the morphological differences and neural mechanisms of attentional control in SCH. In addition, we also explored the associations between current biochemical thyroid status, TSH level and cognition, as well as brain structure and function.

## Materials and Methods

### Participants

A total of 18 patients with SCH were recruited from the Department of Endocrinology, the First Affiliated Hospital of Shantou University. The inclusion criteria were elevated serum TSH levels (≥4.85 mU/L) with normal serum FT3 and FT4 levels (determined at the first laboratory detection of hormone levels), right-handedness, normal vision or good corrected visual acuity, education level ≥ 8 years, age <50 years, and no MRI contraindications. The exclusion criteria were clinical manifestations of past or present hypothyroidism, neuropsychiatric diseases, history of pathology that may affect brain structure and function, or taking thyroid hormone drugs or psychotropic medication in the past, illiteracy, and non-cooperation, or MRI scan contraindications. The control group included 18 demographically matched healthy volunteers from the Logistics Department of the First Affiliated Hospital of Shantou University, who also met the same exclusion criteria as the patients.

All participants completed neuropsychological tests and structural and fMRI scans within 1 week after diagnosis, and the data were included in the further statistical analyses. This study was approved by the institutional review board (i.e., the Medical Ethics Committee of Shantou University). All subjects gave written informed consent before any testing or neuropsychological evaluation.

### Methods

#### Clinical and Neuropsychological Assessment

A chemiluminescence immunoassay (Beckman DXI800 immunoassay system and imported matched reagents, Beckman Co., Brea, CA, USA) was used to determine the serum thyroid hormone level and TSH level of each participant. The Montreal Cognitive Assessment Chinese-revised (MoCA-CR) is an assessment tool commonly used to evaluate the general cognitive status of an individual, such as visuospatial/executive ability, naming, memory, attention, language, abstraction, delayed recall, and orientation using a total of eight subtests (Nasreddine et al., 2005). In this study, the standard for evaluation was used according to the study by Wang et al. (2010) and had a total possible score of 30. Those scoring 26 points or greater were considered to have a normal cognitive function, and those scoring <26 points were considered to indicate a mild cognitive dysfunction. Scores <19 points were used for excluding possible dementia. The MoCA-CR test was marked by two qualified teachers, from the Medical College of Shantou University, who were blind to the aim of the study.

#### fMRI Experimental Design

The Stroop task is a classic neuropsychological test to study selective attention and examines the attention and execution of the brain. In this task, subjects are asked to name a color that conflicts with the meaning of the word. Since word recognition is more automatic and faster than color recognition, the subjects must eliminate the word interference to avoid mistakes, resulting in concentration and reaction time extension. A modified Stroop task with Chinese characters was used in this study.

**Figure 1:**
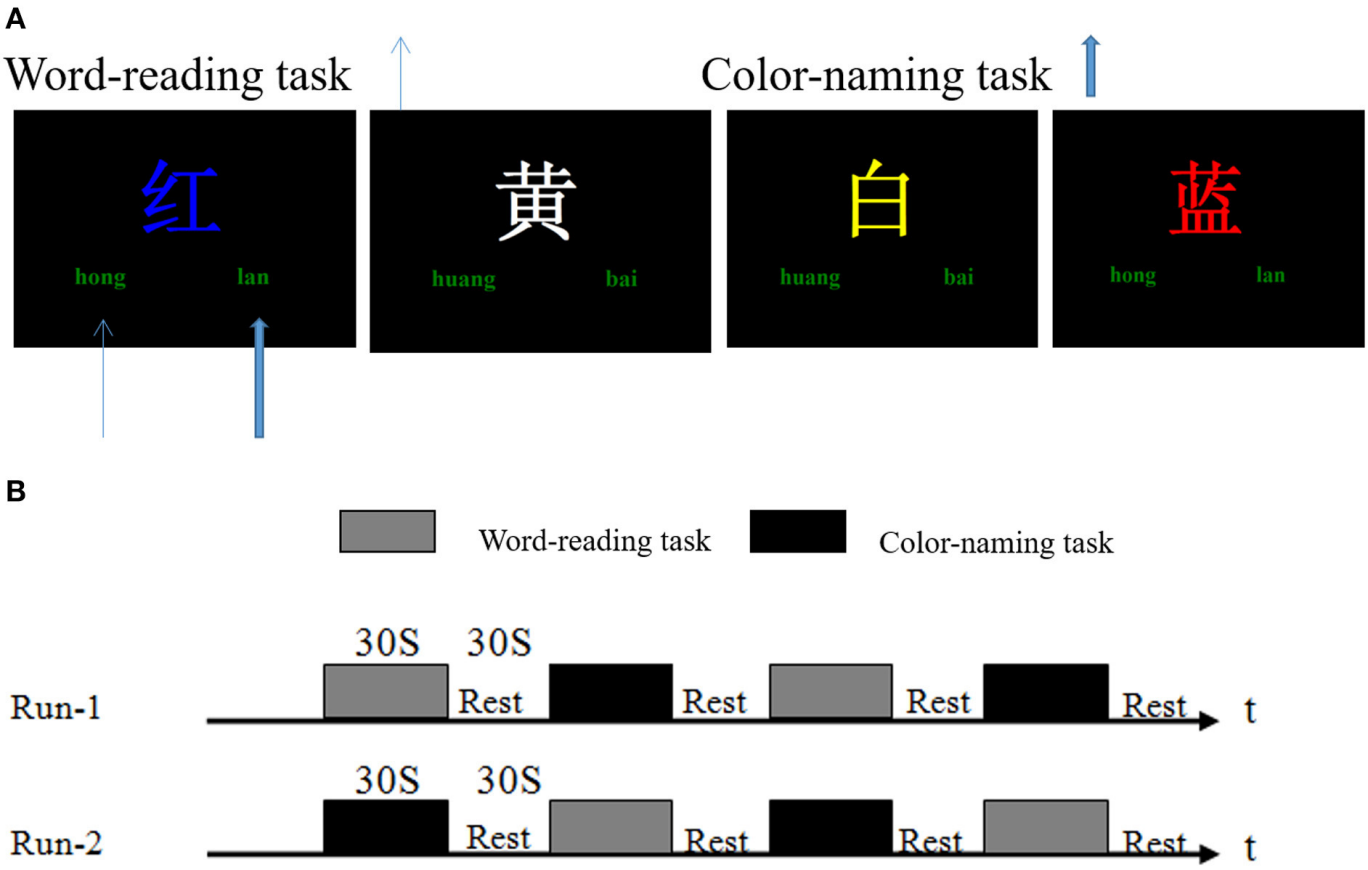
Stroop task and fMRI experimental design. **(A)** Stroop task stimulus presentation. Different written colors of the Chinese characters such as “红(red), 黄(yellow), 蓝(blue), and 白(white)” were randomly presented on the computer screen. “Left click” and “right click” represented the Chinese characters and the color of the word, or the color and the Chinese characters (e.g., in the word reading task, the subjects should left click (“hong,” thin blue arrow), or otherwise right click (“lan,” wide blue arrow) during the color-naming task). **(B)** fMRI experimental design. Two scans were performed for each subject. The task blocks contained word-reading and color-naming tests; each lasting 30 s. The baseline control block (Rest) lasted 30 s, and only a fixation cross was displayed.

A Stroop task began with a hint of 2 s which instructed the participants how to perform the task (word-reading: recognize the Chinese character, or color-naming: judge the color of the Chinese character. Different written colors of the Chinese characters were randomly presented on the computer screen. “Left click” and “right click” represented the Chinese characters and the color of the word, or the color and the Chinese character). After a delay of 1 s, 9 trials were presented to the participants consecutively. Each trial was displayed for 2 s and then followed by a fixation cross for 1 s. During these 3 s, the participants should respond with a left or right thumb to press the right or left button of the response box. Meanwhile, the computer recorded their performance accuracy and reaction time. A baseline control block was also used by showing a fixation cross during the experiment, and the subjects were required to observe the cross at rest (Figure 1A).

One fMRI scan had four epochs. Each epoch consisted of a word-reading task or a color-naming task and followed by a baseline control block. All subjects were given two fMRI scans (Figure 1B). In this study, the E-Prime psychological experiment software system was used to play the stimulus in Windows XP with 640 × 480 resolution on the computer.

#### MRI Data Acquisition

All image acquisitions were collected by experienced neuroradiologists using a 1.5 T MRI scanner (Philips Medical Systems, Best, The Netherlands) with a quadrature coil. The gradient-recalled echo and echo-planar imaging sequence were used to acquire functional images with the following parameters: time of repetition (TR)/time of echo (TE) = 2,000/45 ms, 20 slices (6-mm thick, 1-mm intersection gap), flip angle = 90°, field of view (FOV) = 230 × 230 mm, Matrix = 64 × 64. The following parameters were used to obtain high-quality, three-dimensional (3D) T1-weighted images (T1WI) by a fast low-angle radio frequency pulse sequence: TR/TE = 30/3.0 ms, flip angle = 30°, FOV = 250 × 250 mm, Matrix = 256 × 256, and 120 slices (1.3 mm slice thickness, no gap).

#### Data Analysis

##### VBM Analysis

All MRI structural image data were processed with Statistical Parametric Mapping 8 (SPM8) (http://www.fil.ion.ucl.ac.uk/spm/) and VBM8 toolbox (http://dbm.neuro.uni-jena.de/vbm8), implemented in MATLAB2020a (MathWorks, Natick, MA, USA; http://www.mathworks.com). First, the original images obtained from 3D-T1WI scanning were converted into NifTI file format through MRIConvert software (https://lcni.uoregon.edu/downloads.html). Then, the images were reoriented, normalized to the standard structural template space, and segmented into GM volume (GMV), white matter volume (WMV), and cerebrospinal fluid (CSF). Spatial smoothing was applied with a 6-mm full-width half-maximum (FWHM) Gaussian filter to reduce the remaining interindividual differences and improve the signal-to-noise ratio. Total GM, WM, and CSF volumes of individual patients were obtained based on the segmented images, and total intracranial volume (TIV) was the sum of GM, WM, and CSF volumes.

The brain regions with significant differences (*p* < 0.05, corrected) in regional GMV between groups in VBM analysis were extracted as region of interest (ROI) masks using DPABI (http://www.restfmri.net). These ROI masks were then back-projected to the original images of each patient, and the ROI volume values of each patient were calculated.

##### fMRI Imaging Analysis

The original data were converted from a DICOM format to NifTI using MRIConvert software (https://lcni.uoregon.edu/downloads.html). The MRI data were preprocessed and analyzed using Analysis of Functional NeuroImages (AFNI, https://afni.nimh.nih.gov/). The data preprocessing included slice timing correction to remove any linear drift, motion correction (i.e., only scans with a head motion <2 mm or rotation <2° were used for further analysis), spatial normalization to standard coordinates of the Talairach atlas, and spatial blurring with a 6-mm FWHM Gaussian filter. There were no obvious brain structural abnormalities or signs of cerebral small vessel diseases in any of the participants. Therefore, all fMRI data were reserved for further imaging analysis. Then, the data of each group were averaged and deconvolved according to the experimental task. Differences between the tasks were analyzed with the general linear test.

Intergroup and intragroup analyses were included. For group analysis, the activation map of each group was generated by a correlation analysis based on the direct contrast between the tasks (including word-reading and color-naming tasks) and the control conditions (*p* ≤ 0.05, cluster size ≥ 20 voxels), which were used to locate the ROIs. For each subject, a correlation analysis of the functional data was carried out to get two activation maps and a combined activation map by logical “OR” operation of the maps (*p* ≤ 0.05, cluster size ≥ 20 voxels).

##### Statistical Analysis

The two independent-samples *t*-test was used to compare the clinical and neuropsychological data of both groups. The performance and reaction times of the Stroop task within groups were evaluated with a general linear model for repeated measures ANOVA. The study normalized the GM, WM, and CSF volumes of all subjects by dividing the individual value by the TIV of the respective subject. The two-sample *t*-test was used to compare the normalized GMV (nGMV), normalized WMV (nWMV), and TIV between the two groups.

The framework of the general linear model was used to estimate the differences of the brain GMVs between the SCH group and the control group through the Specify 2nd-level software in SPM. Age, gender, and TIV were used as covariates to enter into the design matrix. The difference was statistically significant with *p* < 0.05 (FDR-corrected). Using xjView software, the clusters with significant differences were superimposed on the T1WI template to generate pseudo-color images showing the GM and WM differences in the whole brain. The coordinates [Montreal Neurological Institute (MNI)], cluster sizes, and *t*-values of the brain regions with statistical significance were observed and recorded.

Pearson's correlation analysis was used to explore the relationship between the regional volumes and the percentage of BOLD signal changes in the PFC, ACC, and precuneus, and the results of the laboratory and MoCA tests, and the accuracy of the Stroop task in patients with SCH. The measurement data are expressed as means ± SD. The *p* < 0.05 was considered statistically significant. The statistical analysis was performed with the Statistical Package for the Social Sciences (SPSS) version 26 for Windows (IBM, Armonk, NY, USA).

## Results

### Demographic and Neuropsychological Data

There were no statistically significant differences between groups in terms of age (*t*-test, *t* = 0.054, *p* = 0.957) and educational background (*t*-test, *t* = 0.289, *p* = 0.774), which suggested that the population characteristics were matched. The TSH levels of patients with SCH were higher than the upper limit of the normal range, with the difference between the two groups being significant (*t*-test, *t* = −12.093, *p* < 0.01). The thyroid hormone levels (FT3 and FT4) were still within the normal range, and there were no significant differences between the two groups (*t*-test, *t* = 0.091, *p* = 0.374; *t* = 1.763, *p* = 0.087).

The cognitive functions as assessed using the MoCA-CR for all subjects showed that there were no significant differences in naming and orientation subtests (*p* > 0.05). However, there were significant differences between the two groups for the other five subsets and total scores (*p* < 0.01). The total scores of patients with SCH were lower than 26, whereas all control subjects scored higher than 26. The general information (age, education level, and hormone levels) and the results of the MoCA performance are summarized in Table 1.

**Table 1:** Clinical information and neuropsychological data of the subjects (mean ± SD).

**Parameter**	**Group**		***T*-value**	***P*-value**
	**Controls (*n* = 18)**	**SCH (*n* = 18)**		
Age (years)	31, 6	31, 6	0.054	0.957
Education level (years)	11, 2	11, 2	0.289	0.768
FT3 (pmol/L)	4.62, 0.46	4.46, 0.63	0.901	0.374
FT4 (pmol/L)	10.41, 1.09	9.72, 1.25	3.564	0.001
TSH (mIU/L)	2.08, 0.74	9.32, 2.43	−12.093	0.000
**MoCA performance**				
Visuospatial/executive	4.89, 0.32	3.78, 0.73	5.890	0.000
Naming	3.00, 0.00	2.83, 0.38	1.844	0.074
Attention	5.78, 0.43	4.94, 0.80	3.888	0.001
Language	2.22, 0.43	1.11, 0.58	6.519	0.000
Abstraction	2.00, 0.00	1.39, 0.50	5.169	0.000
Memory	4.72, 0.46	3.61, 1.10	3.977	0.001
Orientation	5.89, 0.32	5.77, 0.43	0.879	0.836
Total	28.44, 0.70	23.44, 1.15	15.737	0.000

*FT3, free triiodothyronine; FT4, free thyroxine; TSH, thyroid-stimulating hormone; SCH, subclinical hypothyroidism. Normal range: FT3 = 3.6–5.7 pmol/L; FT4 = 9.1–15.4 pmol/L; TSH = 0.51–4.85 mIU/L. Comparison between the SCH group and the control group with the two independent-samples t-test*.

### Behavior Results

The behavior results were mainly analyzed from two aspects, namely, the accuracy and response time of the Stroop task. In a general linear model for repeated measures performed on the Stroop task, performance accuracy, and reaction time were significantly different in both groups [*F*_(1, 34)_ = 52.48, *F*_(1, 34)_ = 132.32, *p* < 0.001]. The performance accuracy of the color-naming task in both groups was lower than that of the word-reading task, and the mean reaction time was longer than that of the word-reading task. The behavioral data indicated that both euthyroid subjects and patients with SCH had Stroop interference effects. The comparisons of the accuracy and reaction time in each task showed significant differences between the two groups, in the word-reading task (*t*-test, *t* = 3.858, *t* = −3.562, *p* < 0.01), and the color-naming task (*t*-test, *t* = 6.573, *t* = −4.675, *p* < 0.01). All these are shown in Table 2.

**Table 2:** Performance accuracy and reaction time of the Stroop task (mean ± SD).

**Groups**	**Word-reading task**		**Color-naming task**	
	**Accuracy, %**	**RT, ms**	**Accuracy, %**	**RT, ms**
Control group (*n* = 18)	98, 1	892, 46	97, 2	1, 000, 55
SCH group (*n* = 18)	96, 2^*^	962, 68^*^	92, 2^*^	1, 151, 124^#^

*RT, reaction time. The intragroup analysis of the two groups using a general linear model (GLM) for repeated measures, and p < 0.05. Interclass analysis of the Stroop tasks between the control group and the SCH group using the two independent-samples t-test*.

* *p ≤ 0.001*,

# *p = 0.002*.

### VBM Results

**Figure 2:**
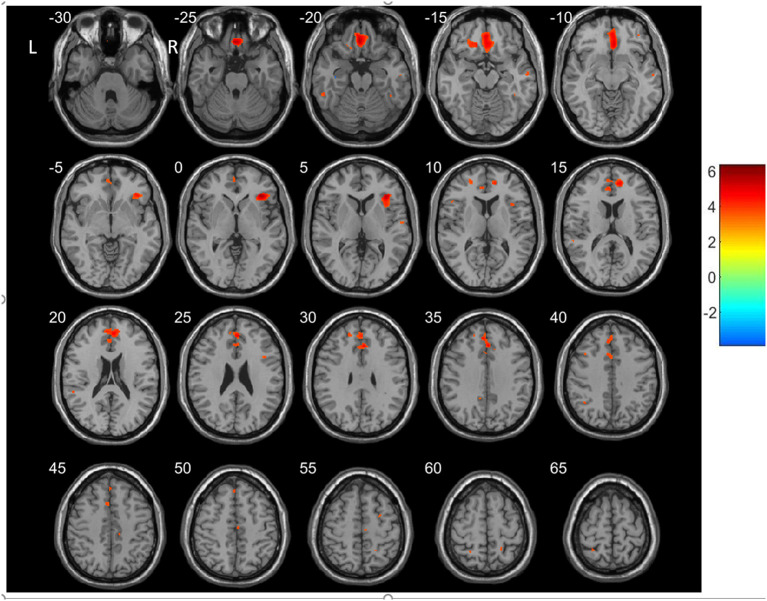
Regions showing decreased gray matter volumes (GMVs) in patients with SCH compared to the controls (*p* < 0.05, FDP-corrected). R, Right; L, left.

For the VBM-based analysis across the whole brain, we found that nGMVs of patients with SCH were significantly less than that of the control group (*p* < 0.05, Table 3). Significantly lower GMVs were found in the bilateral PFCs (including middle, medial, and inferior frontal gyri), cingulate gyrus, precuneus, left middle temporal gyrus, and insula in patients with SCH relative to healthy control subjects (Table 4, Figure 2; *p* < 0.05, FDR-corrected).

**Table 3:** Normalized gray matter volume (nGMV), normalized white matter volume (nWMV), and total intracranial volume (TIV) for two groups (mean ± SD).

**Group**	**nGMV**	**nWMV**	**TIV (ml)**
Control group (*n* = 18)	0.419, 0.025	0.449 ± 0.023	1404.15 ± 125.17
SCH group (*n* = 18)	0.388, 0.020^*^	0.452 ± 0.019	1344.64 ± 166.02

* *nGMV showed a significant difference between the two groups (p < 0.05)*.

**Table 4:** Regions of decreased GMV in SCH compared to controls (control > SCH).

**Anatomic site**	**Hemisphere R/L**	**Cluster size (mm^**3**^)**	**MNI coordinate (mm)**			**Brodmann area (BA)**	***Z*-value**
			**X**	**Y**	**Z**		
Precuneus	L	31	−11	−49	37	BA31	3.23
Rectal/medial frontal gyrus	R/L	1,717	−3	35	−23	BA10/11	4.96
Medial frontal gyrus	R/L	1,373	2	45	−22	BA10	4.04
Cingulate gyrus	R/L	143	−2	15	37	BA32	3.45
Insula	L	43	−56	−37	18	BA13	3.53
Middle temporal gyrus	L	39	−54	−39	18	BA21	3.80
Middle/Inferior frontal gyrus	L	197	−17	30	−15	BA11/47	3.77

*R, right; L, left; BA, Brodmann area; X, Y, and Z represent the maximum intensity point of activation in the MNI coordinate (mm). FDR p < 0.05 and cluster size > 20*.

### fMRI Results

**Figure 3:**
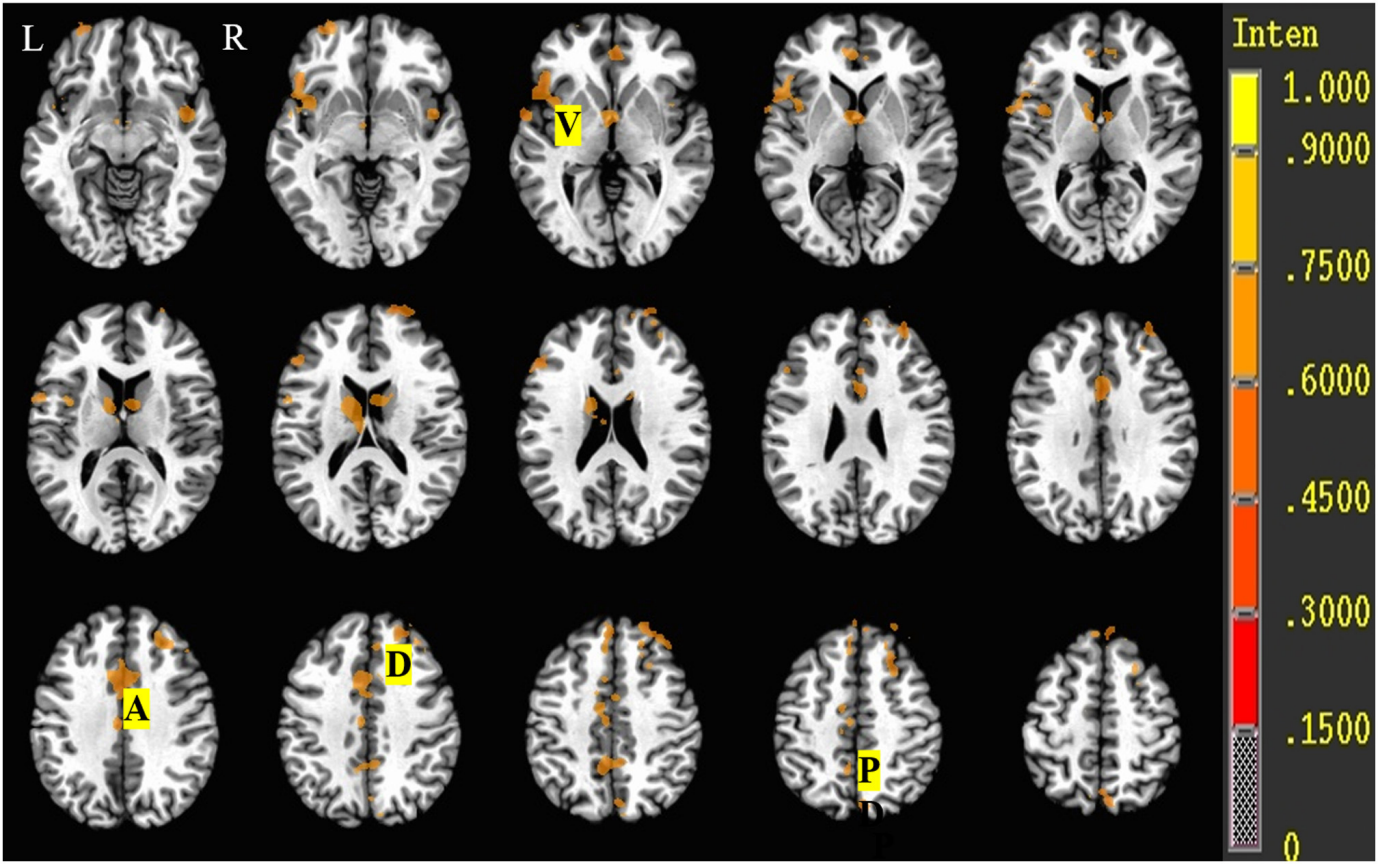
Brain activation during the Stroop task of control and SCH groups. R, Right; L, left; D, dorsolateral prefrontal cortex (DLPFC, BA9/46); V, ventrolateral prefrontal cortex (VLPFC, BA45/47); A, anterior cingulate cortex (ACC, BA24/32); P, posterior cingulate cortex (PCC, BA23/31)/precuneus (BA7).

All the activated areas during the Stroop task in both the SCH group and the control group included PFCs (dorsolateral PFC (DLPFC), superior and middle frontal cortex, BA8/9/46; ventrolateral PFC (VLPFC), inferior frontal cortex, BA45/47), anterior cingulate cortex (ACC, BA24/32), posterior cingulate cortex (PCC, BA23/31), precuneus (BA7), insula (BA13), and the caudate nucleus (Figure 3) and displayed right hemisphere dominance.

**Figure 4:**
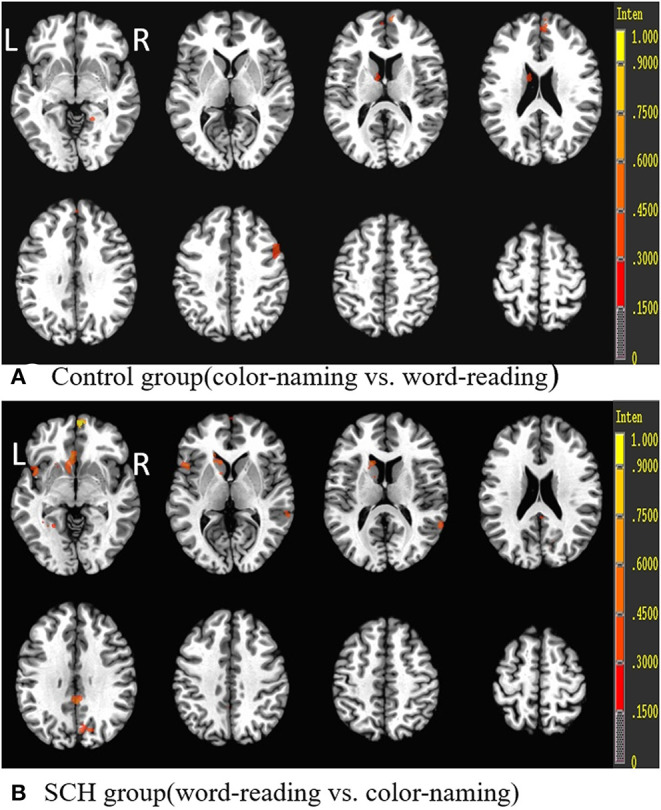
Brain activation differences during the Stroop task (color-naming vs. word-reading or word-reading vs. color-naming) in both groups. R, Right; L, left.

Brain activity data during the Stroop task (i.e., color-naming vs. word-reading task or word-reading vs. color-naming task) of both euthyroid subjects and patients with SCH are shown in Table 5, Figure 4. For euthyroid subjects, more brain areas were activated in the color-naming task, and their activated intensities were higher than those in performing the word-reading task. The bilateral medial frontal gyrus and right middle frontal gyrus (BA8/9/10) and the caudate nucleus were activated in the color-naming task, in contrast to the word-reading task. Although ACC activation was observed in both tasks, there was no significant difference when the tasks were compared. For patients with SCH, when they underwent the color-naming task, the activated brain areas were lower and weaker than the word-naming activations and included only the right insula, left insula/superior temporal cortex, PCC, and lingual cortex. The main attention-related brain areas such as PFC, ACC, and precuneus were not activated but were activated during the word-reading task.

**Table 5:** Brain activation differences during the Stroop task in both groups.

**Stroop task**	**Anatomic site**	**R/L**	**Activation volume (mm^**3**^)**	**Talairach coordinate (mm)**			**BA**	***T*–value**
				**X**	**Y**	**Z**		
Control group (Color naming–word reading)	Medial frontal gyrus	R/L	31	8	59	14	BA10	3.18
	Middle frontal gyrus	R	31	50	11	35	BA8/9	3.011
	Caudate	L	32	−10	−5	10	–	2.45
SCH group (Word reading–color naming)	Medial frontal gyrus	R/L	20	2	59	−4	BA10	6.567
	Superior/middle temporal gyrus	R	24	56	−49	11	BA22/21	8.928
	Insula/inferior frontal gyrus	L	40	−43	11	3	BA13/47	24.2
	Precuneus/PCC	R	23	5	−73	26	BA31	15.76
	ACC	R/L	25	−1	18	−3	BA24	11.08
	Caudate	L	20	−11	15	9	–	13.27

*R, right; L, left; BA, Brodmann area; ACC, anterior cingulate cortex; PCC, posterior cingulate cortex; X, Y, and Z represent the maximum intensity point of activation in the TT coordinate (mm). The p ≤ 0.05 and cluster size ≥ 20*.

**Figure 5:**
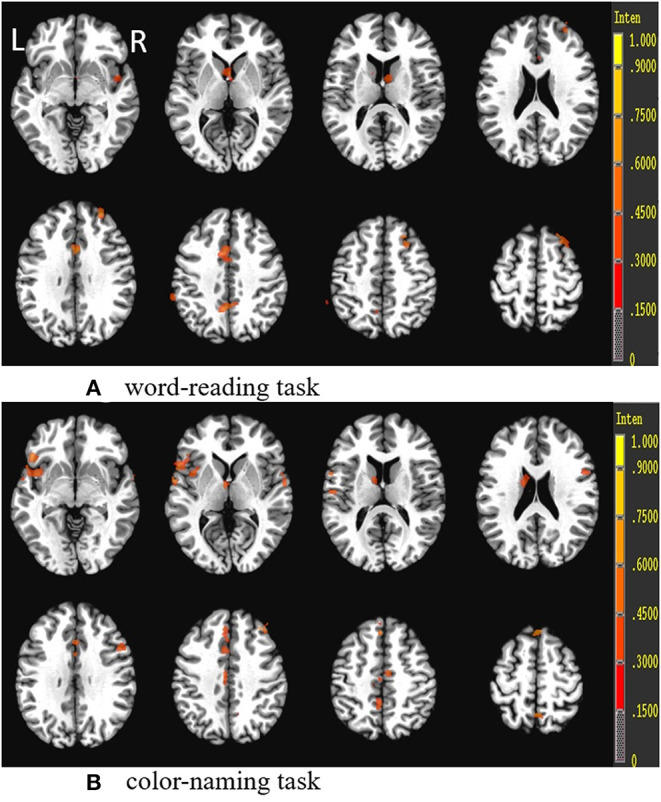
Group differences (control group vs. SCH group) in the Stroop tasks (word-reading task and color-naming task). R, Right; L, left.

The comparison of the activated regions (either word-reading task or color-naming task) demonstrated lower activation in the PFC (mainly DLPFC and VLPFC), parietal lobe (precuneus and inferior parietal lobe), cingulate cortex (ACC and/or PCC), thalamus, and superior/middle temporal cortex in patients with SCH compared to controls (Table 6, Figure 5).

**Table 6:** Brain activation differences of groups in the Stroop task (control group vs. SCH group).

**Stroop task**	**Anatomic site**	**R/L**	**Activation volume (mm^**3**^)**	**Talairach coordinate (mm)**			**BA**	***T*–value**
				**X**	**Y**	**Z**		
Word–reading	Precuneus	R/L	49	−1	−49	38	BA7/31	2.69
	Superior/middle frontal gyrus	R	83	29	17	50	BA6/8/9/10	2.42
	Middle/Inferior frontal gyrus	L	62	−52	23	26	BA9/45/46	2.32
	Cingulate gyrus	R/L	86	2	11	29	BA24/32	2.79
	Insula/Superior temporal gyrus	R	45	44	14	−13	BA38/47	2.96
Color–naming	Superior frontal gyrus	R/L	169	2	41	44	BA6/8	2.15
	DLPFC	L	69	−43	44	17	BA8/9/10/46	2.01
	Cingulate gyrus	R/L	162	−4	−18	43	BA24/31/32	2.27
	Precuneus	R/L	65	−4	−55	29	BA7/31	3.14
	DLPFC	R	36	44	32	32	BA8/9/46	2.22
	Superior temporal gyrus	R	21	59	−6	17	BA22/13	1.97
	Inferior frontal/Insula/Superior temporal gyrus	L	180	−43	17	−1	BA22/47/13	3.31

*R, right; L, left; BA, Brodmann area; DLPFC, dorsolateral prefrontal cortex. X, Y, and Z represent the maximum intensity point of activation in the TT coordinate (mm). The p ≤ 0.05 and cluster size ≥ 20*.

### Correlational Analysis Data in the SCH Group

**Figure 6:**
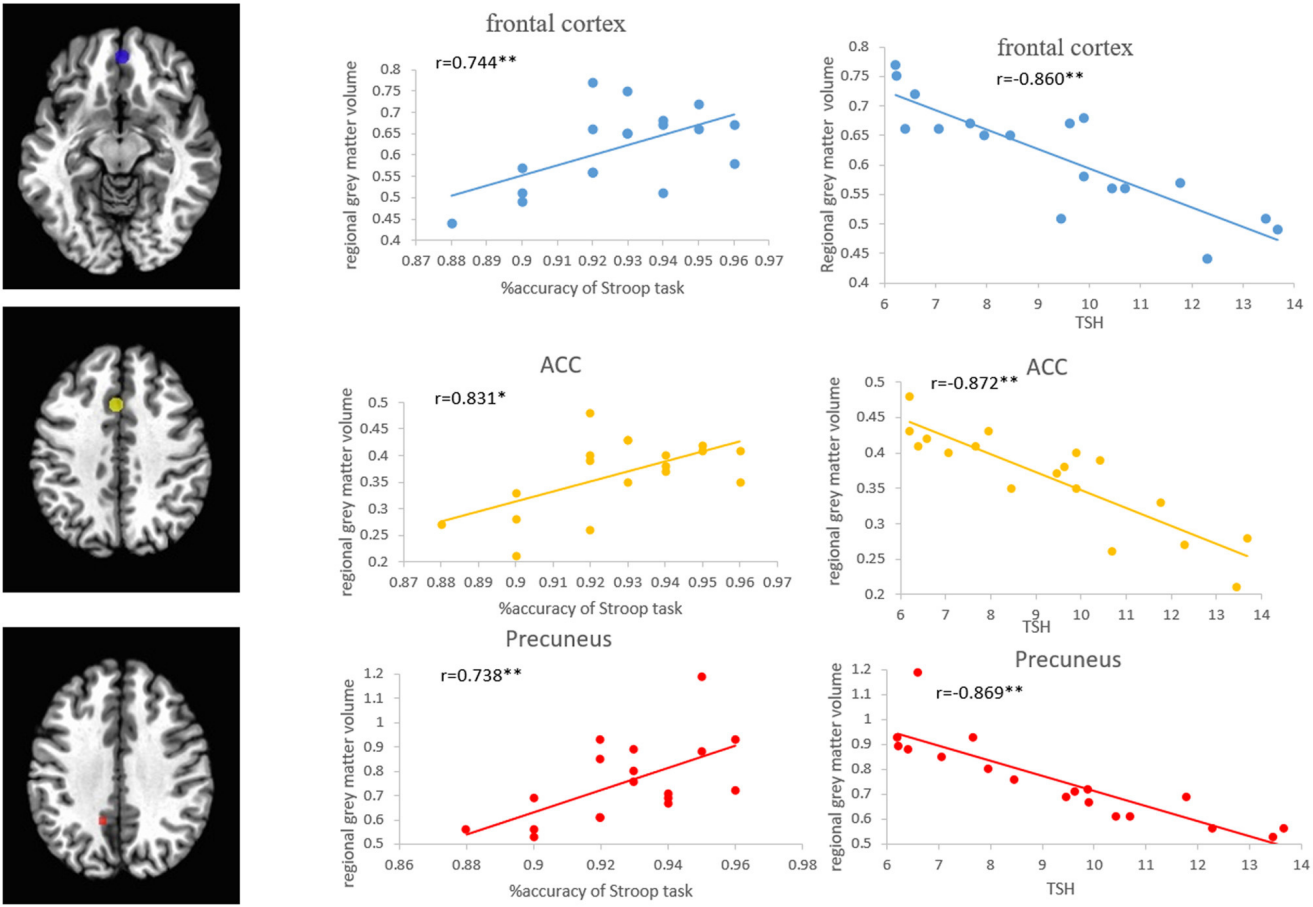
Correlations between the regional GMVs of the frontal cortex, ACC, and precuneus, and the Stroop task and TSH level in patients with SCH. ACC, anterior cingulate cortex; SCH, subclinical hypothyroidism; TSH, thyroid-stimulating hormone.

**Figure 7:**
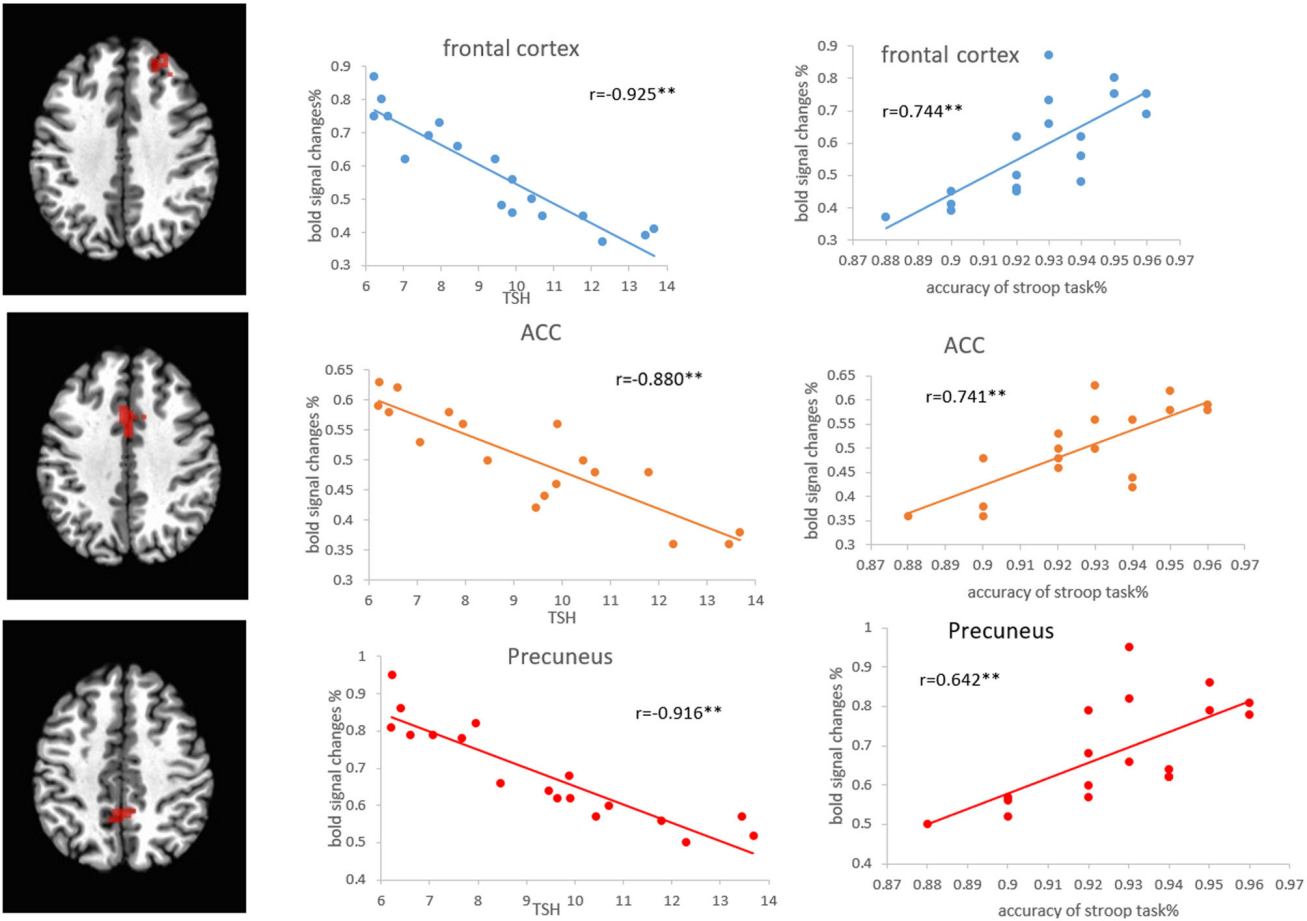
Correlations between the percentage of BOLD signal changes in the frontal cortex, ACC, and precuneus, and the Stroop task and TSH level in patients with SCH. ACC, anterior cingulate cortex; SCH, subclinical hypothyroidism; TSH, thyroid-stimulating hormone.

Pearson's correlation analysis showed that the regional volumes in the PFC, ACC, and precuneus were positively correlated with the accuracy of the Stroop task (*r* = 0.744, *p* < 0.01; *r* = 0.831, *p* < 0.01; *r* = 0.738, *p* < 0.01) and MoCA scores (*r* = 0.771, *p* < 0.01; *r* = 0.763, *p* < 0.01; *r* = 0.764, *p* < 0.01), while negatively correlated with the TSH level (*r* = −0.860, *p* < 0.01; *r* = −0.872, *p* < 0.01; *r* = −0.869, *p* < 0.01) (Figure 6). Pearson's correlation analysis also showed that the percentage of BOLD signal changes in PFC, ACC, and precuneus was positively correlated with the accuracy of Stroop task (*r* = 0.744, *p* < 0.01; *r* = 0.741, *p* < 0.01; *r* = 0.642, *p* < 0.01) and the MoCA scores (*r* = 0.942, *p* < 0.01; *r* = 0.849, *p* < 0.01; *r* = 0.867, *p* < 0.01). There was a significant negative correlation between the TSH levels and the activation intensity of PFC, ACC, and precuneus (*r* = −0.925, *p* < 0.01; *r* = −0.880, *p* < 0.01; *r* = −0.916, *p* < 0.01) (Figure 7). In the SCH group, the Stroop task performance was negatively correlated with the TSH level (*r* = −0.822, *p* < 0.01). Furthermore, the TSH levels were negatively correlated with the MoCA scores (*r* = −0.876, *p* < 0.01). However, there were no correlations between the activation intensity and other serum biomarkers (all *p* > 0.05).

## Discussion

In concordance with our hypothesis, patients with SCH showed neurocognitive and functional brain disorders, as well as structural brain changes, even without significant clinical neurological or psychiatric symptoms. Our study showed that the MoCA total scores of patients with SCH are lower than 26, and the total scores and other 5 subsets are lower than those of the euthyroid subjects, indicating mild cognitive impairment of patients with SCH, in agreement with previous studies (Samuels et al., 2007; Resta et al., 2012).

A modified Stroop task with Chinese characters was used in this study. The results showed that patients with SCH have significantly longer reaction time and lower performance accuracy, which suggests that the patients with SCH may have attentional control function impairment, in agreement with prior studies (Hogervorst et al., 2008; Correia et al., 2009). Through the correlation analysis, we found that patients with higher TSH levels showed negative correlations with Stroop task performance, which suggests that the higher levels of TSH might be harmful to cognition.

Through the VBM analysis, we found significant changes in GMV in patients with SCH. We also demonstrated less GMV in the bilateral PFCs, such as middle, medial, and inferior frontal gyri, cingulate gyrus, precuneus, left middle temporal gyrus, and insula. So far, there have been few VBM studies for patients with hypothyroidism or SCH. Singh et al. used VBM to show diffusion alterations in several regions, such as the left postcentral gyrus, the right precentral gyrus, cerebellum, right inferior and middle frontal gyrus, right inferior occipital gyrus, and right temporal gyrus of patients with hypothyroidism (Singh et al., 2013). The structural changes of the PFC and temporal gyrus were found in both studies. However, decreased hippocampal volume was not found in our study, which is inconsistent with the study of patients with hypothyroid by Cooke et al. (Cooke et al., 2014). This may be related to different data analysis methods, or the SCH had not yet reached the stage when changes in the hippocampal volume occur.

In our fMRI study, a common network consisting of DLPFC, VLPFC, ACC, PCC, temporal gyrus/insula, precuneus, and basal ganglia was activated in the Stroop task. A part of the activated areas overlaps with the brain regions displaying reduced GM or structural abnormalities obtained in our VBM results, especially the PFC, parietal lobe, and cingulate cortex. These are key structures that influence the neural framework of attentional function (Milham et al., 2002; Braem et al., 2017; Fuster, 2019).

Patients with SCH had lower activity and reduced GMV mainly in the PFC, parietal lobe, and cingulate cortex. As is known, the PFCs, especially the middle and inferior frontal gyri, are related to movement, attention, problem-solving ability, and decision-making. The PFC is mainly involved in monitoring and executive control of attentional functions. (Peterson et al., 1999; Leung et al., 2000). DLPFC mainly participates in the attentional control of the Stroop task and may play a role in target operation (Pujol et al., 2001; Herd et al., 2006). The ACC is the pivotal brain area in the conflict monitoring system, which plays an important role in cognitive control (van Veen et al., 2001; Peterson et al., 2002). The specific role of the ACC in cognitive control is to detect conflict and to urge the DLPFC to resolve the conflict (Carter and van-Veen, 2007). Furthermore, the activation level of ACC and DLPFC has been considered to reflect the degree of detection and control of conflict (Nee et al., 2007). Many studies have shown that the parietal cortex is associated with attentional control, usually focusing on its involvement in the visual selection, attention orientation, attention transfer, and stimulus-to-response mappings, which is pivotal for conscious information processing (Smith and Jonides, 1999; Banich et al., 2000). Functional brain imaging in patients with mild cognitive impairment also shows low stimulation in the precuneus, suggesting that the precuneus is essential for conscious information processing (Vogt and Laureys, 2005; Staffen et al., 2012).

Our study indicates that the ACC activation is present in normal subjects when they perform both the word-reading and color-naming tasks. However, no DLPFC and ACC activation are present in patients with SCH when they perform the harder color-naming task. The intergroup analysis of the color-naming task (normal subjects vs. patients with SCH) suggests that the ACC is activated, but the ACC function of patients with SCH is decreased. Due to the dysfunction of the ACC in patients with SCH, they cannot detect conflicts as successfully as normal individuals, which further influences the ability of the PFC to perform cognitive control. Our results suggest that the altered attentional function in patients with SCH might be related to the hypofunction of those areas, especially DLPFC and ACC.

Our intragroup analysis indicates that patients with SCH have dysfunction of various brain areas when they perform the Stroop task, i.e., more brain areas are recruited to accomplish simpler word-reading tasks. As conflicts exceed their control level, patients with SCH cannot accomplish the color-naming task in the same way as healthy individuals, as reflected by the functional and behavioral data. The main attention-related brain areas such as PFC, ACC, and parietal cortex are not activated in patients with SCH when performing the color-naming task, which also suggests that patients with SCH have an abnormal network of cognitive control.

In this study, significant GMV and activity reductions were also found in the temporal gyrus and insula of patients with SCH. The temporal pole has been implicated in memory, attention, and expression of emotional behavior (Blaizot et al., 2010). Moreover, the insula and temporal cortex activation may be related to the processing of the word because studies have found that these regions can be activated when performing visually repetitive word stimuli (Milham et al., 2002). Our study also shows that the basal ganglia (caudate) are activated. Previous studies have found circuits connecting the basal ganglia and cortex, i.e., the basal ganglia mainly project to the thalamus, and the thalamus projects back to the cortex, completing a circuit involving the cortex, striatum, pallidum, and thalamus (Alexander et al., 1986).

We also found that the activation and GMV of the PFC, ACC, and parietal cortex are decreased when patients with SCH perform the Stroop task. These findings are in agreement with the notion of a PFC–ACC–parietal cortex network in conflict detection and resolution (Adleman et al., 2002). Therefore, the attentional function is the interaction between the various structures of the cerebral cortex and subcortical structures, which may be integrated during the formation of attention.

Moreover, it is worth noting that the regional volumes and activation intensity of the PFC, ACC, and precuneus are positively correlated with the performance of the Stroop task and negatively correlated with the TSH levels in the SCH group. The current results indicate that the decreased activation of these regions may be related to a performance decline in SCH. Negative correlations between TSH and Stroop task performance and MoCA scores are also observed in patients with SCH. It has become apparent that patients with SCH with higher TSH levels may be associated with cortical structural abnormalities and cognitive decline.

One limitation of this study is the relatively small study population. Although the sample size of this scale is relatively small, it has systematically been shown that the sensitivity of the BOLD signal is statistically significant in the literature (Wagner, 1999; He et al., 2011). The other limitation is that the research did not study the changes of these patients after drug treatment.

## Conclusion

Our study demonstrates that patients with SCH might have structural and functional changes in brain regions related to attentional control. Moreover, an abnormal PFC–ACC–precuneus circuit might be one of the neural mechanisms responsible for this impaired cognitive control. The PCC, temporal gyrus, insula, and basal ganglia may be involved in the maintenance of attentional control or the link of the PFC–ACC–precuneus circuit. Overall, this study highlights the importance of early diagnosis for SCH, and the higher TSH levels may be a risk factor for abnormalities in the cortex and cognitive decline.

## Data Availability Statement

The original contributions presented in the study are included in the article/supplementary material, further inquiries can be directed to the corresponding author/s.

## Ethics Statement

The study was approved by Medical Ethics Committee of Shantou University (No. 2019104). The patients/participants provided their written informed consent to participate in this study. Written informed consent was obtained from the individual(s) for the publication of any potentially identifiable images or data included in this article.

## Author Contributions

JY was responsible for the conception and the drafting of the manuscript. LX and RG were responsible for the data analysis. MZ and JH were responsible for completing the questionnaire and training. DL and WX were responsible for introducing the subjects and follow-up. ZL was responsible for the data acquisition. SD was responsible for the data evaluation. SM was responsible for the conception and the revising of the manuscript. All authors approved the version to be published and agreed to be responsible for all aspects of the work.

## Funding

This study was financially supported by the grants from the National Natural Science Foundation of China (Grant Nos. 81774395, 82004468, and 81801432), the Natural Science Foundation of Guangdong Province (Grant Nos. 2019A1515011744 and 2018A030307045), the Science and Technology Planning Project of Guangdong Province (Grant No. 2017A020215060), the China Postdoctoral Science Foundation (Grant Nos. 2019M663021 and 2019M652990), the Medical Science and Technology Research Foundation of Guangdong Province of China (Grant Nos. A2020524 and B2020138), the Shantou Technology Bureau Science Foundation of China (Grant Nos. [2017] 119 and [2019] 106), and the Grant for Key Disciplinary Project of Clinical Medicine under the Guangdong High-level University Development Program (Grant No. 002-18120302).

## Conflict of Interest

The authors declare that the research was conducted in the absence of any commercial or financial relationships that could be construed as a potential conflict of interest.

## Publisher's Note

All claims expressed in this article are solely those of the authors and do not necessarily represent those of their affiliated organizations, or those of the publisher, the editors and the reviewers. Any product that may be evaluated in this article, or claim that may be made by its manufacturer, is not guaranteed or endorsed by the publisher.

## References

[B1] Adleman N. E. Menon V. Blasey C. M. White C. D. Warsofsky I. S.. (2002). A developmental fMRI study of the Stroop color-word task. Neuroimage 16, 61–75. 10.1006/nimg.2001.1046 11969318

[B2] Alexander G. E. DeLong M. R. Strick P. (1986). Parallel organization of functionally segregated circuits linking basal ganglia and cortex. Annu. Rev. Neurosci. 9, 357–381. 10.1146/annurev.ne.09.030186.002041 3085570

[B3] Ashburner J. Friston K. J. (2000). Voxel-based morphometry-the methods. Neuroimage 11, 805–821. 10.1006/nimg.2000.0582 10860804

[B4] Banich M. Milham M. Atchley R. Cohen N. J. Wszalek T. Kramer A.. (2000). FMRI studies of Stroop tasks reveal unique roles of anterior and posterior brain system in attentional selection. J. Cogn. Neurosci. 12, 988–1000. 10.1162/08989290051137521 11177419

[B5] Bauer M. Silverman D. H. Schlagenhauf F. London E. D.. (2009). Brain glucose metabolism in hypothyroidism: a positron emission tomography study before and after thyroid hormone replacement therapy. J. Clin. Endocrinol. Metab. 94, 2922–2929. 10.1210/jc.2008-2235 19435829

[B6] Blaizot X. Mansilla F. Insausti A. M.. (2010). The human parahippocampal region. I. temporal pole cytoarchitectonic and MRI correlation. Cereb. Cortex 20, 2198–2212. 10.1093/cercor/bhp289 20064939 PMC2923216

[B7] Braem S. King J. A. Korb F. M. Krebs R. M. Notebaert W. Egner T. (2017). The Role of anterior cingulate cortex in the affective evaluation of conflict. J. Cogn. Neurosci. 29, 137–149. 10.1162/jocn_a_01023 27575278 PMC5341786

[B8] Carter C. S. van-Veen V. (2007). Anterior cingulate cortex and conflict detection: an update of theory and data. Cogn. Affect. Behav. Neurosci. 7, 367–379. 10.3758/CABN.7.4.367 18189010

[B9] Constant E. L. De Volder A. G. Ivanoiu A. Bol A.. (2001). Cerebral blood flow and glucose metabolism in hypothyroidism: a positron emission tomography study. J. Clin. Endocrinol. Metab. 86, 3864–3870. 10.1210/jcem.86.8.7749 11502825

[B10] Cooke G. E. Mullally S. Correia N. O'Mara S. M. Gibney J. (2014). Hippocampal volume is decreased in adults with hypothyroidism. Thyroid 24, 433–440. 10.1089/thy.2013.0058 24205791

[B11] Cooper D. S. Biondi B. (2012). Subclinical thyroid disease. Lancet 379, 1142–1154. 10.1016/S0140-6736(11)60276-6 22273398

[B12] Correia N. Mullally S. Cooke T.un T. K. Phelan N. Feeney J.. (2009). Evidence for a specific defect in hippocampal memory in overt and subclinical hypothyroidism. J. Clin. Endocrinol. Metab. 94, 3789–3797. 10.1210/jc.2008-2702 19584178

[B13] Fuster J. M. (2019). The prefrontal cortex in the neurology clinic. Handb. Clin. Neurol. 163, 3–15. 10.1016/B978-0-12-804281-6.00001-X 31590737

[B14] He X. S. Ma N. Pan Z. L. Wang Z. X. Li N. Zhang X. C.. (2011). Functional magnetic resource imaging assessment of altered brain function in hypothyroidism during working memory processing. Europ. J. Endocrinol. 164, 951–959. 10.1530/EJE-11-0046 21474509

[B15] Herd S. A. Banich M. T. O'Reilly R. C. (2006). Neural mechanisms of cognitive control: an integrative model of stroop task performance and fMRI data. J. Cogn. Neurosci. 18, 22–32. 10.1162/089892906775250012 16417680

[B16] Hogervorst E. Huppert F. Matthews F. E. Brayne C. (2008). Thyroid function and cognitive decline in the MRC cognitive function and ageing study. Psychoneuroendocrinology 33, 1013–1022. 10.1016/j.psyneuen.2008.05.008 18640783

[B17] Kumar M. Modi S. Rana P. Kumar P. Kanwar R. Sekhri T.. (2018). Alteration in intrinsic and extrinsic functional connectivity of resting state networks associated with subclinical hypothyroid. J. Neuroendocrinol. 30:e12587. 10.1111/jne.12587 29504670

[B18] Leung H. C. Skudlarski P. Gatenby J. C. Peterson B. S. Gore J. C. (2000). An event-related functional MRI study of the Stroop color word interference task. Cereb. Cortex 10, 552–560. 10.1093/cercor/10.6.552 10859133

[B19] MacDonald A. W. Cohen J. D. Stenger V. A. Carter C. S. (2000). Dissociating the role of the dorsolateral prefrontal and anterior cingulate cortex in cognitive control. Science 288, 1835–1838. 10.1126/science.288.5472.1835 10846167

[B20] Milham M. P. Erickson K. I. Banich M. T. Kramer A. K. Webb A. Wszalek T.. (2002). Attentional control in the aging brain:insights from an fMRI study of the Stroop task. Brain Cogn. 49, 1277–1296. 10.1006/brcg.2001.1501 12139955

[B21] Nasreddine Z. S. Phillips N. A. Bedirian V. Be'dirian V. Charbonneau S. Whitehead V.. (2005). The Montreal Cognitive Assessment, MoCA: a brief screening tool for mild cognitive impairment. J. Am. Geriatr. Soc. 53, 695–699. 10.1111/j.1532-5415.2005.53221.x 15817019

[B22] Nee D. E. Wager T. D. Jonides J. (2007). Interference resolution: insights from a meta-analysis of neuroimaging tasks. Cogn. Affect. Behav. Neurosci. 7, 1–17. 10.3758/CABN.7.1.1 17598730

[B23] Pasqualetti G. Pagano G. Rengo G. Ferrara N. Monzani F. (2015). Subclinical hypothyroidism and cognitive impairment: systematic review and meta-analysis. J. Clin. Endocrinol. Metab. 100, 4240–4248. 10.1210/jc.2015-2046 26305618

[B24] Peterson B. S. Kane M. J. Alexander G. M. Lacadie C. Skudlarski P. Leung H. C.. (2002). An event-related functional MRI study comparing interference effects in the Simon and Stroop task. Cogn. Brain Res. 13, 427–440. 10.1016/S0926-6410(02)00054-X 11919006

[B25] Peterson B. S. Skudlarski P. Gatenby J. C. Zhang H. Anderson A. W. Gore J. C. (1999). An fMRI study of Stroop word-color interference: evidence for cingulate subregions subserving multiple distributed attentional systems. Biol. Psychiatry 45, 1237–1258. 10.1016/S0006-3223(99)00056-6 10349031

[B26] Pujol J. Vendrell P. Deus J. Junqué C. Bello J. Marti'-Vilalta J. L.. (2001). The effect of medial frontal and posterior parietal emyelinating lesions on stroop interference. Neuroimage 13, 68–75. 10.1006/nimg.2000.0662 11133310

[B27] Quinque E. M. Karger S. Arélin K. Schroeter M. L. Kratzsch J. Villringer A. (2014). Structural and functional MRI study of the brain, cognition and mood in long-term adequately treated Hashimoto's thyroiditis. Psychoneuroendocrinology 42, 188–198. 10.1016/j.psyneuen.2014.01.015 24636515

[B28] Resta F. Triggiani V. Barile G. Benigno M. Suppressa P. Giagulli V. A.. (2012). Subclinical hypothyroidism and cognitive dysfunction in the elderly. Endocrine Metab. Immune Disord. -Drug Targets 12, 260–267. 10.2174/187153012802002875 22385117

[B29] Samuels M. H. Schuff K. G. Carlson N. E. Carello P. Janowsky J. S. (2007). Health status, mood, and cognition in experimentally induced subclinical hypothyroidism. J. Clin. Endocrinol. Metab. 92, 2545–2551. 10.1210/jc.2007-0011 17473069

[B30] Singh S. Modi S. Bagga D. Kaur P. Shankar L. R. Khushu S. (2013). Voxel-based morphometry analysis in hypothyroidism using diffeomorphic anatomic registration via an exponentiated lie algebra algorithm approach. J. Neuroendocrinol. 25, 229–234. 10.1111/jne.12001 23057474

[B31] Smith E. E. Jonides J. (1999). Storage and executive processes in the frontal lobes. Science 283:1657–1661. 10.1126/science.283.5408.1657 10073923

[B32] Staffen W. Ladurner G. Höller Y. Bergmann J. Aichhorn M. Golaszewski S.. (2012). Brain activation disturbance for target detection in patients with mild cognitive impairment: an fMRI study. Neurobiol. Aging 33, 1002.e1–1002.e16. 10.1016/j.neurobiolaging.2011.09.002 21993055

[B33] Taylor S. Kornblum S. Lauber E. (1997). Isolation of specific interference processing in the Stroop task: PET activation studies. Neuroimage 6, 81–92. 10.1006/nimg.1997.0285 9299382

[B34] van Veen V. Cohen J. D. Botvinick M. M. Stenger V. A. Carter C. S. (2001). Anterior cingulate cortex, conflict monitoring, and levels of processing. Neuroimage 14, 1302–1308. 10.1006/nimg.2001.0923 11707086

[B35] Vogt B. A. Laureys S. (2005). Posterior cingulate, precuneal and retrosplenial cortices: cytology and components of the neural network correlates of consciousness. Prog. Brain Res. 150:205–217. 10.1016/S0079-6123(05)50015-3 16186025 PMC2679949

[B36] Wagner A. D. (1999). Working memory contributions to human learning and remembering. Neuron 22, 19–22. 10.1016/S0896-6273(00)80674-1 10027285

[B37] Wang W. Liu D. Gao Z. (2010). Exploration of the cut-off point of the Chinese version of the Montreal cognitive assessment among retired soldiers in Beijing. Chin J. Health Care Med. 4, 271–273.

[B38] Yin J. J. Liao L. M. Luo D. X. Xu K. Ma S. H. Wang Z. X.. (2013). Spatial working memory impairment in subclinical hypothyroidism: an fMRI Study. Neuroendocrinology 97, 260–270. 10.1159/000343201 22986643

[B39] Yuan L. Luan D. Xu X. Yang Q. Huang X. Zhao S.. (2020). Altered attention networks in patients with thyroid dysfunction: a neuropsychological study. Horm. Behav. 121:104714. 10.1016/j.yhbeh.2020.104714 32057820

[B40] Zhang W. Song L. Yin X. Zhang J. Liu C. Wang J.. (2014). Grey matter abnormalities in untreated hyperthyroidism: a voxel-based morphometry study using the DARTEL approach. Eur. J. Radiol. 83, e43–e48. 10.1016/j.ejrad.2013.09.019 24161779

[B41] Zhu D. F. Wang Z. X. Zhang D. R. Pan Z. L.. (2006). fMRI revealed neural substrate for reversible working memory dysfunction in subclinical hypothyroidism. Brain 129, 2923–2930. 10.1093/brain/awl215 16921178

